# Efficacy of Oral Famotidine in Patients Hospitalized With Severe Acute Respiratory Syndrome Coronavirus 2

**DOI:** 10.7759/cureus.22404

**Published:** 2022-02-20

**Authors:** Suraksha Pahwani, Mahesh Kumar, FNU Aperna, Mehak Gul, Darshan Lal, FNU Rakesh, Muhammad Raffey Shabbir, Amber Rizwan

**Affiliations:** 1 Internal Medicine, Jinnah Sindh Medical University, Karachi, PAK; 2 Internal Medicine, Ghulam Muhammad Mahar Medical College, Sukkur, PAK; 3 Internal Medicine, Allama Iqbal Medical College, Lahore, PAK; 4 Family Medicine, Jinnah Postgraduate Medical Centre, Karachi, PAK

**Keywords:** pakistan, anti reflux, management, covid-19, famotidine

## Abstract

Introduction

The clinical benefit of famotidine has been observed in the management of severe acute respiratory syndrome coronavirus 2 (SARS-CoV-2). However, its use in the management of SARS-CoV-2 is intriguing and not well established yet. In this study, we aimed to determine the role of famotidine as adjuvant therapy in improving the outcome of patients hospitalized with coronavirus disease-2019 (COVID-19).

Methods

This two-arm open-label randomized interventional study was conducted in the COVID-19 unit of a tertiary care hospital in Pakistan from December 2020 to September 2021. Patients between the ages of 18 to 65 years, hospitalized with COVID-19 infection, were enrolled in the study. Participants were randomized into two groups. The intervention group received 40 mg oral famotidine daily in addition to the standard care and the control group received standard care as per national guidelines for the treatment of COVID-19 in Pakistan.

Results

Patients admitted with COVID-19 who received famotidine took comparatively fewer days to become symptom-free (8.5 ± 1.7 vs. 9.4 ± 1.9 days, p-value: <0.001) and spent fewer days in hospital (8.6 ± 1.6 vs. 10.3 ± 2.2 days; p-value: <0.0001). However, the overall difference in the need for mechanical ventilation and mortality between the interventional arm and placebo was not significant.

Conclusion

In this study, adding famotidine to standard treatment of COVID-19 was associated with faster clinical recovery and shorter stay in the hospital. However, there was no difference in the need for mechanical ventilation, need for intensive care unit, and overall mortality. Further large-scale studies are needed to understand the role of famotidine in COVID-19 and its mechanism of action in patients with COVID-19.

## Introduction

The novel coronavirus, widely known as the severe acute respiratory syndrome coronavirus 2 (SARS-CoV-2), has infected over 100 million humans worldwide. The first case of coronavirus disease 2019 (COVID-19) was recognized in Wuhan, China, in December 2019 [1]. Due to its highly contagious nature, it quickly spread to other parts of the world, leading to the declaration of a pandemic by the World Health Organization (WHO) in March 2020 [2,3].

The symptoms of COVID-19 have been shown to range from mild fever, cough, headache, and fatigue to a more severe presentation with dyspnea, hemoptysis, and atypical pneumonia [4]. In the most severe cases, COVID-19 can lead to severe acute respiratory syndrome, respiratory failure, and multi-organ shut down with resultant high mortality [4,5]. The diagnosis is confirmed by reverse transcription-polymerase chain reaction (rt-PCR) of samples obtained from oropharyngeal or nasopharyngeal mucosa and is supported by parenchymal changes seen on chest imaging [6,7]. Treatment strategy for mild to moderate COVID-19 involves symptomatic management, including over-the-counter medicines, maintaining hydration, and resting in prone position if dyspneic [8]. Furthermore, using the following agents in the order of preference based on efficacy is recommended in patients at risk of progression to severe COVID-19: ritonavir-boosted nirmatrelvir, sotrovimab, remdesivir, and molnupiravir [8]. The H-2 blocker, famotidine, has been suggested as an FDA-approved drug that could potentially be repurposed for the treatment of COVID-19 [9]. Famotidine has since then been shown to improve patient outcomes and reduce symptom severity in patients acutely ill with COVID-19 [9]. Mather et al. observed that in-hospital death, intubation, and combined death/intubation were significantly lower in the famotidine group than the non-famotidine group [10].

The impact of famotidine on the outcome of COVID-19 is not fully established yet. In this study, we aimed to determine the role of famotidine as adjuvant therapy in improving the outcome of patients hospitalized with COVID-19.

## Materials and methods

This two-arm open-label randomized interventional study was conducted in the COVID-19 unit of a tertiary care hospital in Pakistan from December 2020 to September 2021. Patients aged between 18 and 65 years and hospitalized with COVID-19 infection were enrolled in the study via consecutive convenient non-probability sampling. COVID-19 infection was confirmed via rt-PCR test. Ethical review board approval was taken from Jinnah Sindh Medical University (JSMU/IRB/2020/12). Patients with severe and/or uncontrolled medical conditions with significantly compromised organ function, those who were hospitalized before due to other reasons, patients already on anti-acid therapies, such as histamine receptor blockers or proton pump inhibitors, immunocompromised patients, and pregnant patients were excluded from the study.

The sample size was calculated by using statistical software Epitools [11] with 80% power of the test and 95% confidence interval. Considering 14.5% and 31.1% mortality in COVID-19 patients in the famotidine and non-famotidine groups, respectively [10], the sample size was 89 patients in each group. After taking informed consent and detailed history of the patients, the laboratory values were noted in a self-structured questionnaire. Participants were randomized into two groups in a 1:1 ratio using an online randomizer software, Research Randomizer [12]. The intervention group received 40 mg oral famotidine daily in addition to standard care, and the control group received standard care as per guidelines for the treatment of COVID-19 [8]. Patients were followed for the development of the outcome. The outcomes were defined as the days taken to be symptom-free, the number of days spent in the hospital, the need for intensive care units and/or mechanical ventilation, and death.

SPSS Statistics software (IBM Corp., Armonk, NY), version 23.0, was used for data analysis. For numerical variables, data were expressed as means ± standard deviations. Frequencies and percentages were used for categorical variables. Independent t-test and chi-square were applied to compare variables as appropriate. A p-value less than 0.05 meant that there was a difference between the two groups, and the null hypothesis was void.

## Results

The mean age in the interventional group was 52 ± 11 years and 51 ± 12 years in the placebo arm. Male participants were more in both groups; however, the ratio was equal. Overall demographics and laboratory values were comparable between the two groups (Table 1).

**Table 1:** Demographics and clinical characteristics of participants BPM: breath per minute, CRP: C-reactive protein, LDH: lactate dehydrogenase, NS: nonsignificant, SD: standard deviation

Characteristics (Mean ± SD)	Interventional arm (n=89)	Placebo arm (n=89)	p-value
Age (in years)	52 ± 11	51 ± 12	NS
Male (%)	62	60	NS
Respiratory rate (BPM)	31.7 ± 5.6	31.8 ± 5.5	NS
CRP (mg/L)	120.1 ± 17.2	119.9 ± 17.8	NS
LDH (IU)	324.5 ± 87.2	321.2 ± 88.4	NS
Oxygen saturation (%)	86.5 ± 4.2	86.1 ± 4.3	NS

COVID-19 patients who received famotidine took comparatively fewer days to become symptom-free (8.5 ± 1.7 vs. 9.4 ± 1.9 days, p-value: <0.001) and spent fewer days in hospital (8.6 ± 1.6 vs. 10.3 ± 2.2 days, p-value: <0.0001). The overall difference in the need for mechanical ventilation and mortality between the interventional arm and placebo was not significant (Table 2).

**Table 2:** Outcome of participants in interventional and placebo arm ICU: intensive care unit, NS: nonsignificant, SD: standard deviation

Outcome	Interventional arm (n=89)	Placebo arm (n=89)	p-value
Days taken to become symptom-free (Mean ± SD)	8.5 ± 1.7	9.4 ± 1.9	0.001
Length of hospital stay (Mean ± SD)	8.6 ± 1.6	10.3 ± 2.2	<0.0001
Need for mechanical ventilation (%)	21 (23.5%)	24 (26.9%)	NS
Need for ICU (%)	18 (20.2%)	20 (22.4%)	NS
Overall death (%)	8 (8.9%)	9 (10.11%)	NS

## Discussion

Mixed data regarding the efficacy of famotidine, showing both favorable and unfavorable clinical outcomes in the treatment of COVID-19, have been observed [13]. Our study found better outcomes in terms of clinical recovery and length of hospital stay. However, its effect on the need for ICU and/or mechanical ventilation and mortality was found to be nonsignificant. A retrospective cohort study of hospitalized COVID-19 patients indicated that patients receiving famotidine along with standard treatment had a significantly reduced risk for death or intubation [14]. In contrast, Sun et al. found no significant protective effect of adding famotidine to the standard treatment in reducing the risk of developing serious illness, death, and intubation for COVID-19 patients [15]. 

Famotidine was found to be protective in COVID-19 patients compared with both cimetidine and proton pump inhibitors [16]. There are various possible explanations for why famotidine might work in COVID-19. It may act via its inhibition of histamine signaling and via its arrestin activation [13]. Histamine, bradykinin, and des-arg-bradykinin receptor can all lead to increased endothelial permeability through AKT-1 activation, all of which can lead to pulmonary edema [13,17,18]. The most common cause of death in COVID-19 is due to respiratory failure, caused by acute respiratory distress syndrome (ARDS) [11]. Famotidine may influence the response of the lung due to mast cell degranulation by inhibiting histamine receptors [19]. It may also lower the concentration of cyclic adenosine monophosphate (c-AMP), which may also reduce endothelial permeability [20]. However, the exact mechanism of action is still not postulated.

To the best of our knowledge, this is the first trial in a local setting to study the role of famotidine in improving outcomes of COVID-19. However, the study has its limitations. First, since it was conducted in a single institute, the sample size was less diverse. Second, only one dosing regimen for famotidine was used. Therefore, care should be taken while applying the result to a larger and more diverse population.

## Conclusions

In this study, adding famotidine to standard treatment of COVID-19 was associated with faster clinical recovery and shorter hospital stay. However, there was no difference in the need for mechanical ventilation, the need for ICU, and overall mortality. Further large-scale studies are needed to understand the role of famotidine in COVID-19 and its possible mechanism of action in patients with COVID-19.
